# Deciphering Life’s Mysteries, Courtesy of Marcus Welby, M.D.

**DOI:** 10.7759/cureus.101522

**Published:** 2026-01-14

**Authors:** Diane M Jarrett

**Affiliations:** 1 Department of Family and Preventive Medicine, University of Arkansas for Medical Sciences, Little Rock, USA

**Keywords:** family medicine in history, family medicine in the media, patient-physician relationship, societal changes and family medicine, societal impact

## Abstract

In this editorial, a mixture of memories about the ideal family physician and an examination of the intersection of television and medicine in the late 1960s and early 1970s, the author recalls coming of age while watching the TV program *Marcus Welby, M.D.* Societal changes allowed her to view shows addressing themes that had previously been glossed over or left unmentioned. Although Dr. Welby may have been portrayed unrealistically as a family physician with unlimited time for his patients, he spoke openly and compassionately about many intimate issues. Audience members, including youngsters growing up in pre-internet days, suddenly had access to information about sex and other previously mysterious topics, presented in a straightforward manner by a fictional doctor who inspired trust and communication. For a generation of television viewers, Dr. Welby set the standard for what patients could hope to find in their family physician.

## Editorial

One day in the early 1970s, I sat in my grammar school class listening to the other children speculate about where babies come from. This was before the internet, before social media, and before explicit television programs. For the most part, we kids had no source for authentic information. A classmate was actually shocked by our conversation because his parents had told him (and he believed) that they had found him under a lettuce leaf in the garden.

The rest of the class knew better than that, at least, and I quietly congratulated myself for having an even greater understanding of the enigma, thanks to Dr. Welby.

Many of us in a certain age group remember the 1969-1976 TV series *Marcus Welby, M.D.* with nostalgia: a gray-haired family physician who never rested, his handsome young associate who rode a motorcycle to house calls, and their cheerful nurse, always ready with a medical file or a cup of coffee. Younger generations may recognize Dr. Welby’s name only as a quaint, unrealistic example of what family medicine entails.

Stories involving doctors, real and fictional, have been a staple of film and television for as long as those media have existed. Due to societal norms and censorship prior to the 1960s, these stories were initially limited in their ability to address controversial issues. All of this began changing around 1969, a pivotal year for many reasons. Hundreds of thousands of young people made the trek to Woodstock, and hundreds of thousands more marched on Washington to protest the war in Vietnam. The movie *Midnight Cowboy *won the Academy Award for Best Picture, even though it was X-rated and focused on themes that some years earlier could not possibly have been addressed in an American film. Two more pertinent events occurred in 1969: a new specialty called family practice was approved, and *Marcus Welby, M.D.*, debuted on television.

In the midst of this social upheaval, the Welby show presented provocative material in a non-threatening manner. Medical issues had been discussed on television before, but by the late 1960s, it was possible to explore more mature themes: premarital sex, rape, abortion, drug addiction, teen alcoholism, impotence, vasectomy, depression, and sexual orientation [1,2]. All were potentially problematic topics for TV at the time, but justifiable in terms of what a family physician would encounter in a typical practice.

The Welby producers aimed for authenticity, working with the then-called American Academy of General Practice (AAGP). The AAGP provided a technical advisor to check scripts for accuracy and teach the actors how to interact with their TV patients. A quirky example occurred when Robert Young, who played Welby, called the technical advisor because a script included a line in which Welby asks a female patient if she has any discomfort while urinating. It may be difficult to imagine such delicacy today, but Young (born in 1907) bristled: “I can’t ask a lady if she can urinate. I’m an actor, but ask a beautiful actress if she can urinate? Not me!” They finally agreed he should ask if she felt a burning sensation while using the bathroom [3].

The basic decency that Robert Young projected undoubtedly helped make controversial storylines acceptable in *Marcus Welby, M.D.* Young had been in movies since 1931, often as the boy-next-door type with an engaging smile and quiet charm. In the 1950s, he starred in *Father Knows Best *(1954-1960), a beloved TV series in which he portrayed another idealized figure, a warm and wise father that most of us did not have in real life but wished we did.

The show has left a significant legacy, though it has been assessed in very different ways. In a 2017 article in *Family Medicine*, Susan Y. Lee, MD, coined the term MWM, or “Marcus Welby Moment,” as a positive descriptor of the special bond that patients and doctors can share [4]. Others, however, have noted that Marcus Welby always seemed to have limitless time for each patient, to find a cure within the hour, and to shrug off concerns about payment. The real-life Roger E. Heering, MD (in an unpublished 1971 letter to the AAGP, now available at the American Academy of Family Physicians Foundation’s Center for the History of Family Medicine), groused, “This show really stinks. It portrays the average G.P. about as well as I could portray a Rockette. Marcus Welby apparently sees about one patient a day, if that, plus other incongruities.”

As an adult reflecting on a program from a half-century ago, I consider the differences between a family physician now and then. Dr. Welby had no computers. He relied on books and journals to stay up to date, documenting everything on paper. If he struggled with insurance or reimbursement issues, he never mentioned it. At all times, he was generous and compassionate to his patients, and his long work hours never resulted in expressed feelings of burnout.

But as a child, I thought only that Dr. Welby was the kind of doctor I would like to have. In the opening credits, he looks directly into the camera, pulling back an anesthesia mask as if the camera were the patient. He then gives a warm smile and an encouraging nod. I remember feeling that he was looking into my eyes and that I could trust him to take care of any ailments that befell me. He taught me what to hope for in a patient-physician relationship, realistic or not. Medicine and society have changed, but the need for a compassionate family physician is as meaningful as ever.

Though it is surely coincidental that I pursued a career as a family medicine educator, I am confident that Dr. Welby would be proud. He might even smile and nod.
